# Examining the Factor Structure of Objective Health Literacy and Numeracy Scales: Large-Scale Cross-Sectional Study

**DOI:** 10.2196/71701

**Published:** 2026-01-06

**Authors:** Chihiro Moriishi, Keisuke Takano, Takeyuki Oba, Naoki Konishi, Kentaro Katahira, Kenta Kimura

**Affiliations:** 1 Human Informatics and Interaction Research Institute, National Institute of Advanced Industrial Science and Technology (AIST), 1-1-1 Higashi, Tsukuba, Ibaraki, 305-8566, Japan, 81 80-2202-0737; 2 Faculty of Letters, Hosei University, Chiyoda-ku, Japan

**Keywords:** objective health literacy, objective health numeracy, health-related behaviors, exploratory factor analysis, confirmatory factor analysis

## Abstract

**Background:**

Scales for measuring health literacy and numeracy have been broadly classified into performance-based (objective) and self-reported (subjective) scales. Both types of scales have been widely used in research and practice; however, they are not always consistent and may assess different latent constructs. Furthermore, an increasing number of objective measures have been developed, and it is unclear how many latent factors should be assumed.

**Objective:**

This study aimed to examine the psychometric properties and factor structure of items assessing objective health literacy across multiple scales and to clarify which aspects of objective health literacy would be correlated with subjective measures, as well as health behaviors and lifestyles.

**Methods:**

A total of 5 objective scales (72 items in total) were administered to Japanese-speaking adults (N=16,097; women: 7722/16,097, 48%; mean age 54.89, SD 16.46 years). The analyzed scales included items assessing the numeracy, comprehension, and application of health information, some of which were contextualized for specific diseases, such as diabetes and cancer. Participants’ responses were submitted to exploratory factor analysis, and individual factor scores were calculated to test correlations with subjective health literacy, health behavior, and lifestyle.

**Results:**

Exploratory factor analysis identified 3 factors, which were interpreted as conceptual knowledge, numeracy, and synthesis. The conceptual knowledge factor consisted of items about medical word comprehension. All numeracy items loaded onto the same factor, even when contextualized for different diseases. The synthesis factor was characterized by items assessing the ability to read and understand health-related information and make judgments on it using one’s own knowledge. The identified factors showed high interfactor correlations (*r* values 0.53‐0.64) and small-to-moderate correlations with subjective health literacy (*r* values 0.14‐0.45). Additionally, each factor indicated small positive correlations with healthy diet and nutrition and lower substance use (*r* values 0.17‐0.26).

**Conclusions:**

Our findings suggest that scales of objective health literacy have at least three latent constructs (ie, conceptual knowledge, numeracy, and synthesis) and that disease specificity is not psychometrically prominent. Each factor has some overlap with subjective health literacy, but overall, subjective and objective health literacy should be interpreted as independent constructs, given the small-to-modest correlations.

## Introduction

### Background

Health literacy plays a pivotal role in acquiring and maintaining healthy lifestyles, which help individuals prevent diseases and maintain their well-being [1]. Although the definition of health literacy varies across studies, the core concept refers to the ability of an individual to obtain, process, understand, and use health information and services [2]. This conceptualization covers health numeracy, namely, applying numerical and quantitative reasoning skills to navigate a health care environment, access care, engage in treatment, and make informed health decisions [3]. Empirical studies have demonstrated that lower health literacy, including lower health numeracy, is associated with lower autonomy and self-control in health behaviors as well as negative health outcomes, such as higher older adult mortality, increased emergency and inpatient facility use, lower medication compliance, and lower preventive service use [3,4].

Health literacy assessment has long been a research target, and hundreds of measures have been developed and published over the past 3 decades [5-8]. As Nguyen [6] noted, a typical assessment approach is to ask respondents to self-report about their experience on Likert scales (ie, subjective measurement), whereas it is also common to challenge individuals using standardized test stimuli to evaluate their underlying traits, knowledge, skills, and numeracy [9-12] (ie, objective measurement). For example, the Lipkus Numeracy Scale (henceforth, Lipkus) requires respondents to perform numeracy tests in general (eg, “Imagine that we rolled a fair, six-sided die 1,000 times. Out of 1,000 rolls, how many times do you think the die would come up even (2, 4, or 6)?*”*) [12]. Another typical approach is to assess word comprehension of health-related and medical terms [13]. It is also common to present responders with hypothetical scenarios or visual materials, such as nutrition labels [14,15] or maps of hospitals [16], to assess their ability to read, interpret, and process relevant information. Objective measures have been suggested to be suitable for estimating individual skills guiding actual health behavior [6]—an experimental study showed that individuals with high levels of objective (but not subjective) health literacy were able to critically evaluate health information on websites, which further helped them to choose an appropriate treatment option [17]. In addition, a prospective cohort study on patients with cardiovascular-related diseases showed that the lack of objective health literacy predicted poor refill adherence [18].

In contrast, most subjective measures ask respondents to self-report their perceptions and experiences of handling health information, typically using a Likert scale [6]. The 47-item European Health Literacy Survey Questionnaire (HLS-EU-Q47) [19] is one of the most widely used measures to assess individuals’ perceived abilities to access, understand, appraise, and apply health information (eg, “Finding information on symptoms of illnesses that concern you is...”; respondents indicate from very easy to very difficult) [19]. Another example is the Subjective Numeracy Scale (SNS), which assesses individuals’ beliefs about their skill in performing various mathematical operations (eg, “How good are you at working with fractions?”) and individuals’ preferences regarding the presentation of numerical information (eg, “When reading the newspaper, how helpful do you find tables and graphs that are parts of a story?”) [20]. Subjective measures typically assess individuals’ self-perceived ability to find and understand health-related information as well as their confidence in doing so [17]. Also, some measures cover a wider range of psychological (eg, motivation and self-efficacy) aspects of health literacy [21]. A study suggested that individuals with lower levels of subjective numeracy are less motivated and less confident in numeric tasks [22]. Furthermore, the European Health Literacy Survey showed that subjective (but not objective) health literacy is predictive of self-perceived health [23], which might suggest that subjective measures may be more suited to studying perception and beliefs about health status and behavior.

The objective and subjective measures appeared to tap into the same latent construct, that is, the ability to process health information. However, Waters et al [24] suggested that these 2 types of measures assess conceptually related but psychometrically distinct constructs and that numeracy should be separated from general health literacy. Begoray and Kwan [25] found almost null correlations between objective (word recognition and reading comprehension) and subjective (self-reporting of skills to access and communicate health information) assessments. Marks et al [26] suggested that objective measures may reflect medication knowledge, whereas subjective measures may not. For the associations with health outcomes and behaviors, a systematic review [27] concluded that the evidence is mixed. Several studies observed no differences between performance-based and self-reported health literacy for the associations with relevant health outcomes (eg, diabetes, stroke, and hypertension), whereas others documented objective-subjective discrepancies (eg, for cancer screening use). Hirsh et al [28] noticed that the self-reported disease severity of rheumatoid arthritis was associated with subjective health literacy but not with objective health literacy, including the ability to read and pronounce medical terms.

The possibility that objective and subjective measures assess different constructs of health literacy may make it difficult for researchers and practitioners to determine which type (or both) to include in their assessment batteries. Another challenge when building an assessment battery for health literacy research is that an enormous number of measures have been developed; thus far, there is no clear guidance on which to use and when [8]. Recently, we conducted an exploratory factor analysis of 219 items across 11 subjective measures (encompassing 45 subscales), indicating that dimension reduction was effective, as the items were well explained by 7 latent factors [29].

### Objectives

In this study, we aimed to expand these findings to objective health literacy measures; namely, we conducted an exploratory factor analysis on 5 performance-based measures of health literacy and numeracy (see the *Methods* section for the selection criteria of the analyzed scales), including general and disease-specific (ie, chronic pain, cancer, and diabetes) scales. Through the analyses, we explored how many and what factors would emerge. In addition to the number of factors identified, we were also interested in whether disease-specific items would be recognized as independent factors or factors that reflect common skills and performances regardless of target diseases. Simultaneously, the identified factors were tested for their correlations with lifestyle and health status, as well as subjective health literacy and numeracy, to explore the consistencies and inconsistencies (or validity) with perceived health literacy and behaviors.

## Methods

### Data

Data from a larger longitudinal survey on the health behaviors, psychological characteristics, and lifestyles of Japanese-speaking adults (aged >18 y living in Japan) were used. We used quota sampling to represent the population distribution for age and gender in Japan, and thus, we did not use a survey weight in the analysis. The overarching project (still ongoing) is a 3-year longitudinal study that includes multiple waves with different focuses: wave 1 (N=20,573; early 2023) for physical activity (PA) and psychological characteristics [30] and for mobile health technology use [31], wave 2 (conducted in 2023; 6 mo after wave 1) for changes in PA and digital health behaviors [32], and wave 3 (conducted in early 2024) for health literacy and lifestyle. Wave 3 included both subjective and objective health literacy scales; the psychometric properties of the subjective scales have been reported elsewhere [26]. This study used the wave 3 data (N=16,097; women: 7722/16,097, 48%; mean age 54.89, SD 16.46 years), of which 87% (14,064/16,097) participated in wave 1. As the dropout rate was high, an additional sample of 2033 participants was recruited at wave 3. This addition was for the overarching project but not for this study specifically. Although we could not use quotas in this extra sampling due to the time pressure that we had, we found that the age and gender distributions were similar to those of the general population, so we included this additional sample in the analysis. This study focused exclusively on objective scales. We used data from 5 objective health literacy (or numeracy) scales together with the validation measures of subjective health literacy, health behavior, and lifestyle (refer to the *Measures* section).

### Ethical Considerations

Participants were paid for online panels recruited by a survey firm. Interested individuals followed a link to the survey site, and on the top page, they received study information (written) and provided informed consent to proceed to individual questionnaire pages. Each participant was assigned a study ID, which was used as the key in merging their responses across different waves. No personal information was obtained throughout the study. The study was approved by the Ethics Committee of the National Institute of Advanced Industrial Science and Technology (approval ID: 2022‐1279).

### Measures

#### Objective Health Literacy and Numeracy Scales

We selected the scales for inclusion in this study following published reviews (eg, [8,33,34], including Tavousi et al [8], the latest review on health literacy scales over the past 3 decades when the study was conceptualized, and Nakadai et al [34], a narrative review of the scales available in Japanese). Among the scales listed, we included those that met the following criteria: the scale was available in English or Japanese and could be implemented on a static online survey (ie, did not require audiovisual materials or in-person interactions), and specific instructions and items were available from published articles, supporting materials, or personal correspondence with the authors of the scales. This selection process resulted in four objective health literacy or numeracy scales: the Lipkus [12,35], Newest Vital Sign (NVS) scale [14,15], Functional Health Literacy Scale for Young Adults (funHLS) [13], and Cancer Health Literacy Test (CHLT) scale [16]. An additional database search (Google Scholar and PubMed) identified the Diabetes Health Numeracy (DHN) scale [36], which was eligible for this study. Table 1 summarizes the characteristics of each included scale; most objective health literacy scales are not Likert type. For example, the funHLS presented medical stem terms (eg, caries) and asked participants to indicate the most relevant words for each stem term among 3 response options (eg, virus, bacteria, and fungus). Across the scales, each response was binary coded to represent 1 (correct) and 0 (incorrect), and the total score was calculated for each scale, with higher values indicating higher levels of objective health literacy or numeracy. It should be noted that the current analyses included translated versions of the scales, and responses to some of the items were potentially affected by cultural differences. For example, the NVS and CHLT included items assessing comprehension of food nutrition and prescription medication labels. These stimuli were modified to be familiar to Japanese respondents—particularly for the NVS, the translation and adjustment were conducted rigorously in accordance with the established cross-cultural adaptation guidelines [37,38].

**Table 1.** Overview of the objective health literacy and numeracy scales.

Scale name (abbreviation)	Items, n (Cronbach α)	Test format	Description
Lipkus Numeracy Scale (Lipkus) [12,35]	10 (0.79)	Numeric response questions	Measures the ability to understand and use numeric information, particularly for probability: (eg, “Imagine that we rolled a fair, six-sided die 1,000 times. Of 1,000 rolls, how many times do you think the die would come up even (2, 4, or 6)”)?
Newest Vital Sign (NVS) scale [14,15]	6 (0.63)	Numeric response questions and open-ended questions	Measures comprehension, numeracy, and application and evaluation skills. Responders are presented with a nutrition label of ice cream, from which they are required to extract necessary information for calculation (eg, “If you eat the entire container, how many calories will you eat?”) and evaluation (eg, “Pretend that you are allergic to the following substances: penicillin, peanuts, latex gloves, and bee stings. Is it safe for you to eat this ice cream?”)
Functional Health Literacy Scale for Young Adults (funHLS) [13]	19 (0.93)	Multiple choice questions	Measures knowledge and comprehension of health-related and medical terms. Responders are presented with stem words, for each of which they are asked to indicate the most relevant among 3 response options (eg, stem=caries: response options=virus, bacteria, and fungus).
Diabetes Health Numeracy (DHN) scale [36]	7 (0.85)	Multiple choice questions	Measures numeracy skills, contextualized for diabetes (eg, “If you walk for about 30 minutes you can burn 100 calories. If you want to burn 150 calories, how long do you have to walk?”). Several items tap into interpretation skills (eg, “read a table about diagnostic criteria for diabetes and indicate the stage of an example patient”).
Cancer Health Literacy Test scale (CHLT) [16]	30 (0.85)	Multiple choice questions	Measures knowledge (eg, “Which is the highest in calories and protein? – French fries, cheeseburger, hard-boiled egg”), comprehension skills (eg, “In people who develop oral cancers, 25% of these cases occur in the tongue. Oral cancer occurs in the tongue*...”*), and their synthesis, contextualized for cancer.

#### Subjective Health Literacy Scale

The HLS-EU-Q47 [19,39] was used to assess subjective health literacy. The HLS-EU-Q47 and other self-report scales (see below) were used as validation measures to test for correlations with objective health literacy measures. The HLS-EU-Q47 measures 4 information-processing competencies (ie, how easy it is to access, understand, appraise, and apply health information) for 3 health-relevant domains (ie, health care, disease prevention, and health promotion). Participants indicated how applicable each item was to them using a 4-point scale (1=very easy and 4=very difficult). For ease of interpretation, each item was reverse scored, with higher values indicating higher health literacy levels, and the total score was normalized to a range between 0 and 50 using the following formula: (mean−1)×(50/3). This scale has shown good reliability in the current data (Cronbach α=0.97).

#### Subjective Health Numeracy Scale

The SNS was used to assess subjective health numeracy levels [20]. The SNS measures one’s perceived ability to perform mathematical tasks (eg, How good are you at working with fractions?) and preferences for the use of numerical (vs prose) information (eg, When reading the newspaper, how helpful do you find tables and graphs that are parts of a story?). Participants indicated how applicable each item was to them using a 4-point scale (1=not good at all, not helpful at all; 4=very good, very helpful). This scale has shown good reliability in the current data (Cronbach α=0.75).

#### Physical Activity

The International Physical Activity Questionnaire Short Form [40,41] was used to assess PA levels. Respondents were asked to indicate the number of days and minutes per day spent walking, engaging in moderate-intensity activities, and engaging in vigorous-intensity activities. We did not use sedentary time for the current analyses. The weighted sum of the reported durations was calculated across the 3 activity categories, representing the total PA in the form of metabolic equivalents (METs hours per week). According to the Ministry of Health, Labour and Welfare in Japan, the recommended amount is 23 METs hours per week or higher for adults aged <65 years and 10 METs hours per week for older people [42].

#### Quality of Life and Health State

Quality of life (QoL) and health status were assessed using the 5-level EuroQol 5-Dimension (EQ-5D) version [43]. Participants indicated their health status by selecting the most appropriate statement (ie, no problems to extreme problems) for the following five dimensions: mobility, self-care, usual activities, pain or discomfort, and anxiety or depression. Participants’ responses were combined into a 5-digit code, which was then converted into a numerical QoL score. The QoL score ranges from −0.025 to 1, where a negative value signifies a condition worse than death, 0 represents a state equivalent to death, and 1 denotes the highest possible health utility. At the end of the EQ-5D questions, participants were asked to rate their health status using a visual analog scale, ranging from 0 to 100, with 0 representing the worst health condition they could imagine and 100 representing the best health condition they could imagine.

#### Health-Related Lifestyles

The Short Multidimensional Inventory Lifestyle Evaluation (SMILE; [44]) consists of 45 items covering seven domains of health-related lifestyles: diet and nutrition, substance use, PA, strategies to deal with stress, sleep pattern, social support, and environmental exposure. Items asking about the use of illegal drugs (ie, items 10 and 11) were excluded to adhere to the ethics standards of the administering survey firm, and the remaining 43 items were used in the survey. Participants rated each item on a 4-point scale (1=always and 4=not at all). Summed scores were calculated for each domain, whereas items were reverse scored (with higher values indicating healthier lifestyles). The global score (sum of the 7 domains) demonstrated good internal consistency in the current data (Cronbach α=0.88).

### Statistical Analysis

An exploratory factor analysis was conducted on the 5 objective health literacy and numeracy scales. We excluded from the analysis (1) an item (funHLS12) exhibiting high correlations with other items (*r* values 0.72‐0.80) and (2) 5 items to which >90% of participants responded correctly (ie, items 2, 4, 14, and 27 of the CHLT and item 5 of the Lipkus). The final dataset consisted of 66 items. As each item was binary scored (correct vs incorrect), polychoric correlations were calculated and used in factor analysis. The number of factors was determined based on the reduction in eigenvalues (ie, a scree plot), as well as on the interpretability of the identified factors. Exploratory factor analysis was conducted on randomly sampled 70% of the data (n=11,268), and the remaining 30% (n=4829) was used for confirmatory factor analysis as testing data. Before factor analyses, each dataset was tested with the Kaiser-Meyer-Olkin sampling adequacy measure (≥0.8; Kaiser 1970; [45]) and Bartlett sphericity test (*P*≤.05; [46]). Confirmatory factor analysis was conducted with maximum likelihood estimation to replicate the factor structure obtained in the exploratory factor analysis. Our focus was on the goodness of fit of the model to the data, evaluated by the following indices: chi-square [47], comparative fit index [48], root mean square error of approximation [47,49], and the standardized root mean square residual [47]. For each factor, items with factor loadings of 0.40 or greater (a commonly used threshold for identifying meaningful loadings; eg, see [50]) were interpreted and were used to calculate a factor score (as the mean of raw item scores). For each factor, items with factor loadings of 0.40 or greater were interpreted and were used to calculate a factor score (as the mean of raw item scores). These factor scores were tested for correlations with validation measures (ie, subjective health literacy and numeracy scales, PA, QoL, health status, and health-related lifestyles). All analyses were performed using R (version 4.3.3; R Foundation for Statistical Computing). The *factanal* function was used for the exploratory factor analysis, and the *cfa* function of the *lavaan* package [51] was used for the confirmatory factor analysis.

## Results

### Descriptive Information

Table 2 presents the descriptive statistics. For the objective measures, the mean scores were comparable to those reported in previous studies—for example, in the general Japanese population (Lipkus, mean 9.6) [35], an Italian population-based sample (NVS, mean 4.1) [52], and a sample from the United States (CHLT, mean 22.3) [16]. The total score on the HLS-EU-Q47 was slightly higher than that reported among Japanese people (mean 25.3) but lower than that reported among Europeans in the literature (mean 33.8) [39].

**Table 2.** Descriptive statistics (n=16,097).

Variable	Values
Age (y), mean (SD)	54.89 (16.46)
Gender (women), n (%)	7722 (48)
Objective health literacy and numeracy scales, mean (SD)	
Lipkus^a^	7.80 (2.34)
NVS^b^	3.50 (1.69)
funHLS^c^	14.14 (5.21)
DHN^d^	5.38 (2.09)
CHLT^e^	24.67 (4.89)
Subjective health literacy and numeracy scales, mean (SD)	
HLS-EU-Q47^f^	28.23 (8.07)
SNS^g^	3.24 (0.67)

a Lipkus: Lipkus Numeracy Scale.

b NVS: Newest Vital Sign scale.

c funHLS: Functional Health Literacy Scale for Young Adults.

d DHN: Diabetes Health Numeracy scale.

e CHLT: Cancer Health Literacy Test scale.

f HLS-EU-Q47: 47-item European Health Literacy Survey Questionnaire. A general health literacy index score comprising all items was standardized on a metric between 0 and 50, using the following formula: (mean − 1) × (50/3).

g SNS: Subjective Numeracy Scale.

### Exploratory Factor Analysis

**Figure 1.**
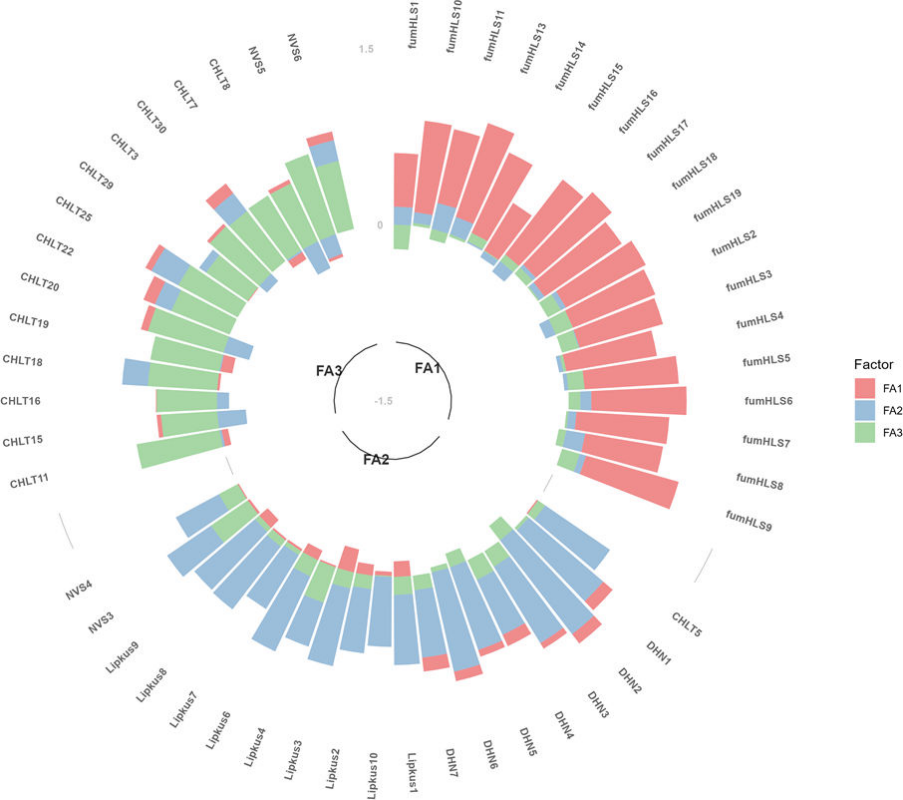
Items’ factor loadings on each factor. CHLT: Cancer Health Literacy Test scale; DHN: Diabetes Health Numeracy scale; FA: factor; funHLS: Functional Health Literacy Scale for Young Adults; Lipkus: Lipkus Numeracy Scale; NVS: Newest Vital Sign scale.

The factor analysis performed on 66 items across 5 scales revealed eigenvalues of 16.43, 3.09, 1.93, and 1.59 for the 1- to 4-factor solutions. The reduction in the eigenvalue supported the 3-factor solution, with explained variances of 0.16, 0.15, and 0.10 for the 3 factors (total explained variance: 0.41). Additionally, the 3-factor solution had good interpretability; the factor loadings are visualized in Figure 1, which confirms that no items had double or triple loadings. The exact factor loadings for each item are listed in Table S1 in Multimedia Appendix 1. Table 3 summarizes the characteristics of each factor.

**Table 3.** Interpretations of identified factors.

Factor^a^ and item with a factor loading of 0.40 or higher		Example item
FA1 (conceptual knowledge)		
funHLS^b^ (items 1‐11 and 13‐19)		funHLS 6: “Indicate the most relevant word for *Vitamin C*. Response options*: Vegetables, Fat, Grain, and I Don’t know.*”
FA2 (numeracy)		
Lipkus^c^ (items 1‐4, 6‐10)		Lipkus 6: “If Person A’s risk of getting a disease is 1% in ten years, and person B’s risk is double that of A’s, what is B’s risk?”
NVS^d^ (items 3 and 4)		NVS 3: “Your doctor advises you to reduce the amount of saturated fat in your diet. You usually have 42 g of saturated fat each day, which includes 1 serving of ice cream. If you stop eating ice cream, how many grams of saturated fat would you be consuming each day?”
DHN^e^ (items 1‐7)		DHN 2: “A male diabetic patient weighs 80 kilograms (kg). The doctor advised this patient to lose 10% of his weight. How much weight does this patient need to lose?”
CHLT^f^ (item 5)		CHLT 5: “In people who develop oral cancers, 25% of these cases occur in the tongue. Oral cancer occurs in the tongue...”
FA3 (synthesis)		
NVS (items 5 and 6)		NVS 5: “Pretend that you are allergic to the following substances: Penicillin, peanuts, latex gloves, and bee stings. Is it safe for you to eat this ice cream?”
CHLT (items 3, 7‐8, 11, 15‐16, 18‐20, 22, 25, and 29‐30)		CHLT 18: “An appointment card says not to eat or drink anything 9 hours prior to the appointment. Sally has an appointment at 11:15 a.m. on Friday. What time should she stop eating or drinking?”

a The means and SDs of each item as well as their factor loadings are provided in the supplementary materials in Multimedia Appendix 1.

b funHLS: Functional Health Literacy Scale for Young Adults.

c Lipkus: Lipkus scale.

d NVS: Newest Vital Sign scale.

e DHN: Diabetes Health Numeracy scale.

f CHLT: Cancer Health Literacy Test scale.

Factor 1 (FA1) consisted exclusively of items from the funHLS, which asked participants to indicate the word most relevant to a stem (medical) word. Items from the funHLS assess word comprehension and knowledge about diseases and symptoms that young adults often experience, as well as nutrition, diet, and human biology. All items of the funHLS showed loadings of >0.40 on FA1. Although the funHLS items covered a range of topics (eg, caries, depression, and BMI), most items loaded on the same factor, and items from other scales were not included in FA1. This factor could be interpreted as a conceptual knowledge of health-related and medical terms in general (ie, not limited to a particular disease or health condition); however, it is still possible that the factor may reflect the unique test format, as the other scales require binary (true-false) responses or numeric responses, for example, to calculate a probability or health risk.

Factor 2 (FA2) included items from 4 of the 5 analyzed scales (ie, Lipkus, NVS, DHN, and CHLT), representing performance-based health numeracy in general (eg, “Imagine that we rolled a fair, six-sided die 1,000 times. Out of 1,000 rolls, how many times do you think the die would come up even (2, 4, or 6)?”). These 4 scales target different populations—Lipkus was designed for the general population, whereas the other 3 were contextualized for particular diseases and health conditions (DHN for diabetes and CHLT for cancer). The test format also differed across the 4 scales; the CHLT used multiple-choice questions, whereas the NVS and DHN included numeric response questions. These results suggest that the items assessing performance-based numeracy correlate well with each other, regardless of heterogeneity in the target diseases and test format.

Factor 3 (FA3) included items from 2 scales, the NVS and CHLT, which assess the ability to process and synthesize health-related information. For example, item 5 of the NVS concerns abstract reasoning, integrating reading, comprehending, and interpreting skills as applied to material with health content [15]. Respondents were presented with a hypothetical nutrition label of ice cream and asked to judge whether the ice cream would be safe if the respondents were allergic to the indicated substances. Similarly, many of the items loaded onto FA3 required respondents to comprehend and synthesize the presented information (eg, the nutrition label) to make the correct response. Items from the CHLT are contextualized in a daily cancer patient routine at a clinic (eg, instructions for the use of medicines and reading a floor map of a hospital), assessing respondents’ knowledge, numeracy, navigation, and synthesis [16]. Therefore, compared to FA1 (word comprehension and knowledge) and FA2 (numeracy), FA3 is distinguished in that it broadly measures higher order skills that require the synthesis of multiple skills (eg, reading, comprehension, and interpretation) to apply in a daily health context.

### Confirmatory Factor Analysis

The testing dataset was found suitable for factor analysis: Kaiser-Meyer-Olkin=0.98 and the Bartlett Test of Sphericity, *P*≤.001. We built a confirmatory factor analysis model with the 3 factors identified through exploratory factor analysis. This model showed an excellent fit to the testing data, χ^2^_1271_=7015.7, comparative fit index=0.97, root mean square error of approximation=0.03, and standardized root mean square residual=0.05, which reassures that the analyzed scales can be reduced to the 3 factors.

### Correlation Analysis

The 3 identified factors were tested for their correlations with subjective health literacy and numeracy, as well as with health status and lifestyle (Table 4). Each correlation was interpreted for magnitude but not for statistical significance, given the large sample size of the analyzed dataset. The Cohen guideline was used, with *r*=0.10, 0.30, and 0.50 being interpreted as small, moderate, and large effects, respectively [53,54]. FA1 to FA3 showed large interfactor correlations. However, these factors showed small-to-moderate correlations with the HLS-EU-Q47 (subjective health literacy), SNS (subjective numeracy), and SMILE (subscales of diet, nutrition, and substance use). Moreover, FA1 and FA2 showed small correlations with SMILE sleep and social support (*r* values 0.10‐0.13). None of the factors showed interpretable size correlations with the International Physical Activity Questionnaire Short Form (total PA) or EQ-5D (QoL and subjective health) scores. The 2 subjective measures, the HLS-EU-Q47 and SNS, presented stronger correlations with the SMILE subscales, except for substance use, than FA1 to FA3.

**Table 4.** Correlations between each factor and comprehensive health status.

	Values, mean (SD)	FA1^a^	FA2^b^	FA3^c^	HLS-EU^d^	SNS^e^
FA1	0.74 (0.27)	—^f^	—	—	—	—
FA2	0.75 (0.24)	0.63	—	—	—	—
FA3	0.80 (0.19)	0.53	0.64	—	—	—
HLS-EU-Q47	28.23 (8.07)	0.24	0.19	0.14	—	—
SNS	3.24 (0.67)	0.33	0.45	0.32	—	—
Total physical activity (METs^g^ hours per week)	34.20 (55.21)	−0.00	−0.02	−0.05	0.09	0.06
EQ-5D^h^ quality of life	0.82 (0.14)	−0.01	0.03	0.02	0.10	0.07
EQ-5D health status	76.17 (17.59)	0.05	0.06	0.02	0.18	0.12
SMILE^i^ diet	2.88 (0.49)	0.26	0.24	0.19	0.33	0.27
SMILE substance use	3.29 (0.82)	0.21	0.17	0.20	0.08	0.07
SMILE physical activity	2.29 (0.62)	0.05	0.05	−0.02	0.24	0.19
SMILE stress management	2.40 (0.48)	0.11	0.09	0.02	0.32	0.21
SMILE sleep	2.77 (0.58)	0.10	0.11	0.05	0.23	0.17
SMILE social support	2.59 (0.63)	0.13	0.11	0.06	0.31	0.23
SMILE environment	2.44 (0.53)	0.04	0.03	−0.02	0.16	0.13

a FA1: factor 1 (conceptual knowledge).

b FA2: factor 2 (numeracy).

c FA3: factor 3 (synthesis).

d HLS-EU-Q47: 47-item European Health Literacy Survey Questionnaire. A general health literacy index score comprising all items was standardized on a metric between 0 and 50, using the following formula: (mean − 1) × (50/3).

e SNS: Subjective Numeracy Scale.

f Not available.

g MET: metabolic equivalent.

h EQ-5L: EuroQol 5-dimension.

i SMILE: Short Multidimensional Inventory Lifestyle Evaluation.

## Discussion

### Principal Findings

This study examined the factor structure of the multiobjective health literacy and numeracy scales among Japanese-speaking adults. Specifically, we explored how many factors would emerge in the pool of 72 items extracted from 5 scales, with or without being contextualized for specific diseases. The exploratory factor analysis indicated that the items could be categorized into three factors: performance-based conceptual knowledge (FA1), numeracy (FA2), and synthesis (FA3).

Most funHLS items loaded on FA1, assessing the conceptual knowledge of health-related and medical terms. FA2 consisted of items from 4 scales targeting people with different health conditions and diseases that typically assess their ability to perform mathematical calculations. The NVS and CHLT items not included in FA1 were identified as FA3, which required the synthesis of multiple skills to handle health information, such as reading, knowledge, navigation, and interpretation skills, to provide a correct response. A correlation analysis indicated that all factors had weak correlations with subjective health literacy, moderate correlations with subjective health numeracy, and weak correlations with lifestyle (eg, diet, nutrition, and substance use). Lifestyles concerning sleep and social support demonstrated small correlations only with FA1 and FA2 but not with FA3.

In line with Altin et al [9] and Wu et al [55], we observed small-to-moderate correlations between the 3 factors and the subjective scales (ie, HLS-EU-Q47 and SNS). Furthermore, the 3 identified factors were highly correlated with each other, yet were recognized as independent factors. These findings echo Waters et al’s [24] argument—health literacy and numeracy are related but distinct constructs, each of which can be psychometrically divided into performance-based (objective) and self-reported (subjective) constructs. Another important point is that our analysis did not identify disease-specific factors, although we included cancer- and diabetes-specific items in the item pool. Therefore, it is plausible to assume that the 3 identified factors—conceptual knowledge, numeracy, and synthesis—form a common basis for processing health information in general. Health literacy covers a range of skills from basic to advanced levels. Basic skills include reading and writing (ie, literacy), which allow individuals to function effectively in everyday situations. These skills serve as a foundation for more advanced ones, for example, extracting information, deriving meaning from different sources of communication, and applying new information to changing circumstances [1]. We assume a similar hierarchical structure for the identified 3 factors, which may explain the interfactor correlations; that is, synthesis represents higher order skills that require more basic ones, such as numeracy and knowledge, along with other cognitive and literacy skills (eg, reading, comprehension, and interpretation).

Regarding the associations with health behaviors and lifestyles, each factor presented small correlations with diet and substance use but not with PA. Some overlaps were noticed at the item content level; for example, the NVS includes items about caloric calculation as well as reading and interpreting a nutrition label, whereas the SMILE asks how often respondents eat high-calorie sweet or fatty foods and how frequently they check the food ingredient labels. A similar association was found in patients with diabetes; performance-based numeracy is positively correlated with a healthy diet [56]. These findings suggest that skills and abilities assessed by objective measures underlie perceived health behaviors (eg, individuals are able to read and interpret ingredient labels and check them regularly when shopping for food). However, the size of the correlations was modest, and the results should be interpreted carefully, particularly for the practical significance.

Compared with objective measures, subjective measures demonstrated overall larger correlations with health behaviors and lifestyles. The conceptual knowledge and numeracy factors (FA1 and FA2) had small correlations with sleep and social support of the SMILE (*r* values 0.11‐0.13) but subjective health literacy (HLS-EU-Q47) and numeracy (SNS) presented slightly larger correlations with sleep and nutrition (*r* values 0.17‐0.33) as well as with other subscales (eg, PA, *r*=0.24; stress management, *r*=0.33). Higher levels of objective health literacy are thought to be associated with an inclination to behave in a manner that is beneficial to one’s own and others’ health (eg, choosing beneficial treatments for a disease) [17]. However, subjective health literacy may share even larger variance with the perception of health behaviors; that is, how people perceive their ability to process health information may overlap with how they believe to behave in a context where their health matters. It is too early to conclude that subjective measures are more suited for studying health behaviors based only on the correlations found in this study. Instead, it is fair to argue that objective and subjective measures reflect different psychological processes, and further research is warranted to clarify which type (or both) of health literacy measure is associated with actual health behaviors that can be assessed using sensors and devices, such as accelerometers for PA.

### Limitations

This study has several methodological limitations. First, the item pool was neither exhaustive nor comprehensive. Importantly, we did not include Test of Functional Health Literacy in Adults (TOFHLA) [10] and Rapid Estimate of Adult Literacy in Medicine (REALM) [11], which are the most widely used objective measures, because of language and cultural differences (all materials had to be in the Japanese language) and technical limitations of the survey platform (audio-visual recording could not be implemented). Both tools are closely bound to the English language (or even to the culture and health care system of the country where the scales were developed). For example, the REALM evaluates whether respondents pronounce medical terms correctly, and the TOFHLA assesses the ability to read and understand health-related materials contextualized in the US health care system. Yet, our analyses covered the scales and items conceptually overlapping with the REALM and TOFHLA; the funHLS is a word recognition test for medical terms, the CHLT and NVS assess reading comprehension of texts and tables, and the Lipkus evaluates numerical ability. However, we acknowledge that the exact items of the REALM and TOFHLA were not included here, and this may affect the interpretation of the results, particularly for the generalizability of the study findings. Furthermore, it is highly likely that the results of the factor analysis and subsequent analyses might differ if the item pool were expanded. Second, the exploratory factor analysis showed that the 3-factor structure explained less than half of the total item variance. A possible explanation is that measurement invariance might not be assumed in subgroups of participants as the data covered a diverse range of people in terms of demographics and other psychosocial variables. Different factor structures could be found across participants with different backgrounds, which should be clarified in future research. Third, participants were recruited using quota sampling to match the known population distribution in Japan for age and gender. Quota sampling is useful to ensure broad coverage of different groups and to prevent overrepresentation of a particular group in data. However, this approach is known to be vulnerable to sampling bias within a subgroup, which could be addressed by the use of self-weighted sampling if the cost of random sampling does not matter. Fourth, diagnostic information on physical or mental disorders was not collected. Testing patients with a particular disease or disorder was out of our focus, as we set a community sample as our target population. Health literacy is essential in maintaining one’s health and preventing future diseases. However, it is important to widen the focus to include patient care and disease management, for which health literacy and assessments are highly relevant. Fifth, convenient self-reporting tools were used to assess PA and lifestyle habits. Health behaviors can be assessed using wearable devices and e-diaries (eg, food recordings), which may allow for a more reliable estimation of healthy lifestyles [57]. It was technically impossible for us to use device- or sensor-based assessments, given the sample size of this study, but objective assessment tools could be considered when a focused sample is the research target.

### Conclusions

Despite these limitations, our findings contribute to the psychometric evidence base of objective health literacy and numeracy scales. The results of the exploratory factor analysis identified 3 factors—conceptual knowledge, numeracy, and synthesis—among 66 items from 5 scales, independent of disease specificity and different contextualizations of the items. These 3 factors showed marginal correlations with subjective measures of health literacy and numeracy, highlighting the distinction between performance-based and self-reported assessment approaches [58]. Researchers and practitioners should be aware that self-report measures do not always reflect the skills and abilities reflected in performance on tests assessing conceptual knowledge, numeracy, and more integrated information processing skills. In other words, both subjective and objective measures should be considered if one wishes to assess different aspects of health literacy. In general, subjective measures are easier to administer and less cognitively demanding [6,7]; also, these measures are more suitable for assessing meta-cognitive, emotional, or motivational aspects of health literacy rather than knowledge and numeracy [22,27]. However, self-reported measures are vulnerable to social desirability and other biases owing to health beliefs [20], which may reduce the accuracy of assessing health information skills [9]. In contrast, objective measures are less affected by response biases [6,17] but may feel like examinations and evoke a sense of shame and stigma. This aspect is particularly relevant for individuals feeling uncomfortable with examinations and not confident in their skills (eg, test anxiety). Also, objective measures often cover a limited, highly contextualized range of skills [6]. Given these advantages and disadvantages, it is not readily possible to uniformly determine the best measures to assess health literacy. It is important for individual researchers to be aware of what aspects of health literacy they want to assess, which then helps them select appropriate scales and items in line with their objectives.

## Supplementary material

10.2196/71701 Multimedia Appendix 1 The means and SDs of each item as well as their factor loadings.

## Abbreviations

CHLT: Cancer Health Literacy Test
DHN: Diabetes Health Numeracy
EQ-5D: EuroQol 5-Dimension
funHLS: Functional Health Literacy Scale for Young Adults
HLS-EU-Q47: 47-item European Health Literacy Survey Questionnaire
Lipkus: Lipkus Numeracy Scale
MET: metabolic equivalent
NVS: Newest Vital Sign
PA: physical activity
QoL: quality of life
REALM: Rapid Estimate of Adult Literacy in Medicine
SMILE: Short Multidimensional Inventory Lifestyle Evaluation
SNS: Subjective Numeracy Scale
TOFHLA: Test of Functional Health Literacy in Adults
